# The change of myopic prevalence in children and adolescents before and after COVID-19 pandemic in Suqian, China

**DOI:** 10.1371/journal.pone.0262166

**Published:** 2022-03-21

**Authors:** Hongyan Chen, Ya Liao, Wen Zhou, Lei Dong, Wei Wang, Xiaojuan Wang

**Affiliations:** 1 Department of Ophthalmology, The Affiliated Xuzhou Municipal Hospital of Xuzhou Medical university, Xuzhou, Jiangsu Province, China; 2 Xuzhou Medical University, Xuzhou, Jiangsu Province, China; 3 The Primary and Middle School Health Care Center in Suqian, Suqian, Jiangsu Province, China; 4 School of Public Health, Xuzhou Medical University, Xuzhou, Jiangsu Province, China; National Yang-Ming University Hospital, TAIWAN

## Abstract

**Purpose:**

The aim of this study was to investigate the change of myopic prevalence in students with different demographic characteristics before and after the COVID-19 pandemic in Suqian, China.

**Methods:**

A retrospective, cross-sectional study was conducted. Student data from 52 schools in 2019 and 2020 were collected from the electronic medical records database through cluster sampling. Ophthalmic examinations were conducted on students from September to December in 2019 and 2020. Measurements of uncorrected visual acuity (UCVA) and noncycloplegic autorefraction were included to obtain the spherical equivalent refraction (SER) and prevalence of myopia. The difference in the rate of myopia and SER of students ages 6 to 18 with various demographic characteristics was compared between the two years.

**Results:**

Records from 118,479 students in 2019 and the 121,881 students in 2020 were obtained. In 2019 and 2020, the prevalence of overall myopia increased from 43.1% to 48.9% (5.8 percentage point), and a substantial shift in myopic rate occurred in grades 4 to 6 (6.9 percentage point). The change in the prevalence of myopia in girls (5.9 percentage point) was approximately equal to that in boys (5.8 percentage point) and it was more common in rural students (5.9 percentage point) than in urban students (5.1 percentage point). The prevalence of low myopia increased more in children, and the prevalence of moderate myopia increased more in adolescents. The mean spherical equivalent refraction (SER) (-1.34±2.03 D) was lower in 2020 than in 2019 (-1.16±1.92 D), while SER decreased mainly at ages 7 to 15. The SER presented myopic status at the age of 9 (-0.55±1.26 D in 2019, -0.71±1.42 D in 2020), and attained moderate myopia at the age of 15 (-3.06±2.41 D in 2019, -3.22±2.40 D in 2020).

**Conclusions:**

After the COVID-19 pandemic, myopia increased in this population with variable rates of increase in different demographic groups. The change of myopia in children was comparatively greater than that in adolescents. Therefore, we should take measures to prevent and control the development of myopia after the COVID-19 pandemic, especially for younger students.

## Introduction

COVID-19 is an infectious disease [1], that rapidly spread around the world during 2020. Home quarantine was an effective way to combat the spread of COVID-19 in China [2], but it imposed a large social burden because people stopped working and travel was restricted. In addition, the Chinese Ministry of Education proposed "online teaching" as a strategy to limit viral transmission during the pandemic. However, this method not only decreased students’ out-door activities but also increased their screen time [3, 4]. In 2020, during the COVID-19 pandemic, approximately 220 million children and adolescents completed their courses through online learning [5].

The prevalence of myopia is notably severe in China [6, 7], and is expected to increase [8]. Visual impairment occurs with complications of high myopia, such as retinal detachment, cataracts, glaucoma, and others, which negatively affect individual livelihoods and increase socioeconomic burdens [9, 10]. Less outdoor activity and more screen time are common risk factors for the development of myopia [11]. Therefore, the assessment of myopia is particularly important to pay attention to after the COVID-19 pandemic [12, 13].

The Chinese government considers the prevention and control of myopia a serious problem [14]. The Primary and Middle School Health Care Center in Suqian (PMSHCCS) is a branch unit under the guidance of the Affiliated Xuzhou Municipal Hospital of Xuzhou Medical University (Xuzhou No. 1 People’s Hospital) and is responsible for screening the vision of local children and adolescents. Suqian is located in northern Jiangsu Province, eastern China, and its economy is relatively developed compared with the cities of western China. According to the Suqian Education Website, schools in Suqian started online teaching on February 1, 2020. Approximately 98.7% of students attended classes online in some schools. Starting on April 1st, the students successively entered schools, and online teaching lasted for approximately 2 months [15–17]. We hypothesize that this may have affected the development of myopia in these students. This retrospective, cross-sectional study analyzed data from 2019 and 2020 to investigate the difference in myopia amongst various demographic groups to reveal the change in prevalence of myopia after the COVID-19 pandemic.

## Methods

### Subjects

This retrospective, cross-sectional study was initiated by Xuzhou No. 1 People’s Hospital in August 2021. The primary data originated from the electronic medical records database of the PMSHCCS, which was collected from September to December in both 2019 and 2020. Schools were initially selected through exclusion criteria and the final sample was obtained through cluster sampling. Inclusion criteria were as follows: schools had been screened twice in consecutive years, and the screening interval needed to be 10–12 months. There are more than 400 primary and secondary schools in Suqian, and the data of more than 200 schools are in PMSHCCS database. The database includes schools in 4 districts of Suqian, including the Muyang district, Sihong district, Siyang district and Suqian district. Ten to fifteen schools were included in each district through cluster sampling. Students were from 35 rural schools and 17 urban schools. These 52 schools were also selected in 2020, so some of the students in 2019 were examined again in 2020. Education is compulsory for children and adolescents from grades 1 to 12 in China; therefore, some schools in this study included both elementary and middle school students, while some studies only included one of them. The age of the students in this study refers to their age on the date of vision screening. Data collected included demographic parameters, as well as visual acuity and spherical equivalent refraction (SER) of students aged 6 to 18 years in Suqian in 2019 and 2020.

The study adhered to the tenets of the Declaration of Helsinki and was approved by the ethics board of Xuzhou No. 1 People’s Hospital, Xuzhou, China. (No: xyy11[2021]059). The PMSHCCS was responsible for on-site work and contacting schools in Suqian. Due to the large sample size of the study, the PMSHCCS informed the subjects about this retrospective study by text message. Oral consent was acquired from students’ parents or guardians to approve the use of their data for research. Those who declined to participate were excluded. All the above practices were approved by the ethics committee.

### Demographic parameters and ophthalmic examination

Vision screening was conducted from September to December in both 2019 and 2020. Students’ demographic information was obtained by admission information before ophthalmic examination, including name, sex, age and region. All subjects were informed that they would undergo ophthalmic examination at least 3 days in advance through a text message to their parents or guardian, to ensure that students would not wear their contact lens or glasses on the examination date.

Ophthalmic examination included distance visual acuity measurement and noncycloplegic refraction. Students were tested in the order of grade, class and student number. Uncorrected visual acuity (UCVA), was performed by 5 professional optometrists. Before visual examination, the examiner asked if the student had ever worn an ortho-K lens to correct myopia, which was recorded as being linked to symptoms of myopia. UCVA was monocularly recorded in logMAR scores by using the standard logarithmic visual acuity chart with a 5-point recording at 5 m. Noncycloplegic refractive error was examined by 4 professional optometrists using 2 instruments of autorefractometry (autorefractor KR-8900, Topcon, Tokyo, Japan), and the spherical equivalent refraction (SER) was calculated as the spherical refractive error + 1/2 of the cylindrical refractive error. All subjects were measured 3 times. If the difference in the SER between any two tests was more than 0.50 D, a remeasurement was required. For the students who failed to receive the examination on the examination day, we organized them to receive the examination in a designated hospital later.

### Definition of myopia

In our study, myopia was defined as UCVA (logMAR) < 5.0 and SER of ≤ −0.50 dioptres (D) in either eye [18, 19], or receiving corneal refractive therapy, such as wearing ortho-K lenses. The following categories of myopia were designated: mild myopia (≤ -0.50 D to >-3.00 D), moderate myopia (≤−3.00D to >-6.00 D), and high myopia (≤ −6.00 D) [20]; other conditions were defined as nonmyopia.

### Statistical analysis

Statistical analysis was performed using IBM SPSS Statistics 23.0, Microsoft Excel 2010, and SPSS AU. We excluded data with incomplete information, such as missing UCVA, SER, or demographic characteristics. We analyzed the de-identified data removing students’ name and number. Continuous variables and data with a normal distribution are represented as the mean ± standard deviation, and count data are represented as the frequency (rate). Pearson’s chi-squared test was used to evaluate differences in the prevalence of myopia with various demographic parameters, and the Cochran-Armitage test for trends was used to evaluate the association between the prevalence of myopia and age. A two-proportion z test was used to assess differences in myopic rate in 2019 and 2020. The SER of the right eye was recorded, and the difference in SER was compared using two independent sample tests. Pearson’s correlation analysis was used to reveal the relation between SER and age. P values lower than 0.05 were considered statistically significant.

## Results

### General information

The distribution of populations with different demographic parameters is listed in Table 1. In the 52 schools in this study population, a total of 123,681 (coverage of 97.0%) and 122,417 (coverage of 99.7%) students underwent ophthalmic examinations in 2019 and 2020, respectively. Of these, data from 5,202 children in 2019 and 536 children in 2020 were excluded because of incomplete information, as well as students less than 6 or greater than 18 years of age. In total, 118,479 (efficiency of 95.8%) and 121,881 (efficiency of 99.6%) students in 2019 and 2020 were included in this study, respectively.

**Table 1:** The distribution of populations with different demographic parameters.

Categories		Students(*n*)	
		2019	2020
**Total**		118,479	121,881
**Age**			
**6**		9,792	9,977
**7**		14,052	13,119
**8**		13,473	14,135
**9**		13,910	14,204
**10**		14,220	14,285
**11**		12,672	13,717
**12**		12,079	11,788
**13**		10,313	10,284
**14**		8,167	9,325
**15**		4,345	4,978
**16**		2,511	2,963
**17**		2,016	2,429
**18**		929	677
**Grade**			
**Primary school**	**1–3**	41,280	39,729
	**4–6**	40,065	42,246
**Junior school**		29,075	30,559
**Senior school**		8,059	9,347
**Gender**			
**Male**		63,557	64,920
**Female**		54,922	56,961
**Region**			
**Urban**		57,270	62,425
**Rural**		61,209	59,456

### Myopic prevalence

The prevalence of myopia is presented in Table 2. In 2019 and 2020, the total prevalence of myopia increased by grade (15.8% to 87.5% in 2019, 20.4% to 88.4% in 2020, *P*<0.05). The prevalence of myopia in girls was higher than in boys (46.9% and 52.8% in girls, 39.8% and 45.6% in boys, *P*<0.05), and higher in urban areas than in rural areas (49.6% and 54.7% in urban students, 37.0% and 42.9% in rural students, *P*<0.05). Furthermore, the prevalence of myopia was 35.3% and 73.6% in primary and junior school students in 2020, respectively, which was significantly higher than the 28.9% and 70.6% in 2019 (*P*<0.05). The prevalence of myopia in different genders and regions was notably higher in 2020 than in 2019 (*P*<0.05).

**Table 2:** Demographic factors associated with myopic prevalence in all participants.

Variables		2019		2020		*z*	*P* ^c^
		*n*	%	*n*	%		
**Total**		51,063	43.1	59,643	48.9	-28.70	<0.01
**Grade**							
**Primary school**	**1–3**	6,535	15.8	8,098	20.4	-16.84	<0.01
	**4–6**	16,946	42.3	20,801	49.2	-19.98	<0.01
**Junior school**		20,529	70.6	22,477	73.6	-8.02	<0.01
**Senior school**		7,053	87.5	8,267	88.4	-1.88	0.06
***χ*** ^***2***^		27,765.23		25,791.95			
***P*** ^a^		<0.01		<0.01			
**Gender**							
**Male**		25,303	39.8	29,592	45.6	-20.91	<0.01
**Female**		25,760	46.9	30,051	52.8	-19.58	<0.01
***χ*** ^***2***^		604.15		625.04			
***P*** ^b^		<0.01		<0.01			
**Region**							
**Urban**		28,388	49.6	34,163	54.7	-17.85	<0.01
**Rural**		22,675	37.0	25,480	42.9	-20.60	<0.01
***χ*** ^***2***^		1,892.20		1,717.39			
***P*** ^b^		<0.01		<0.01			

Values are presented as number (%).

^a^ Comparison underwent Cochran—Armitage test for trends.

^b^ Comparison underwent Pearson’s chi-squared test.

^c^ Comparison underwent Two-proportions z test.

**Fig 1:**
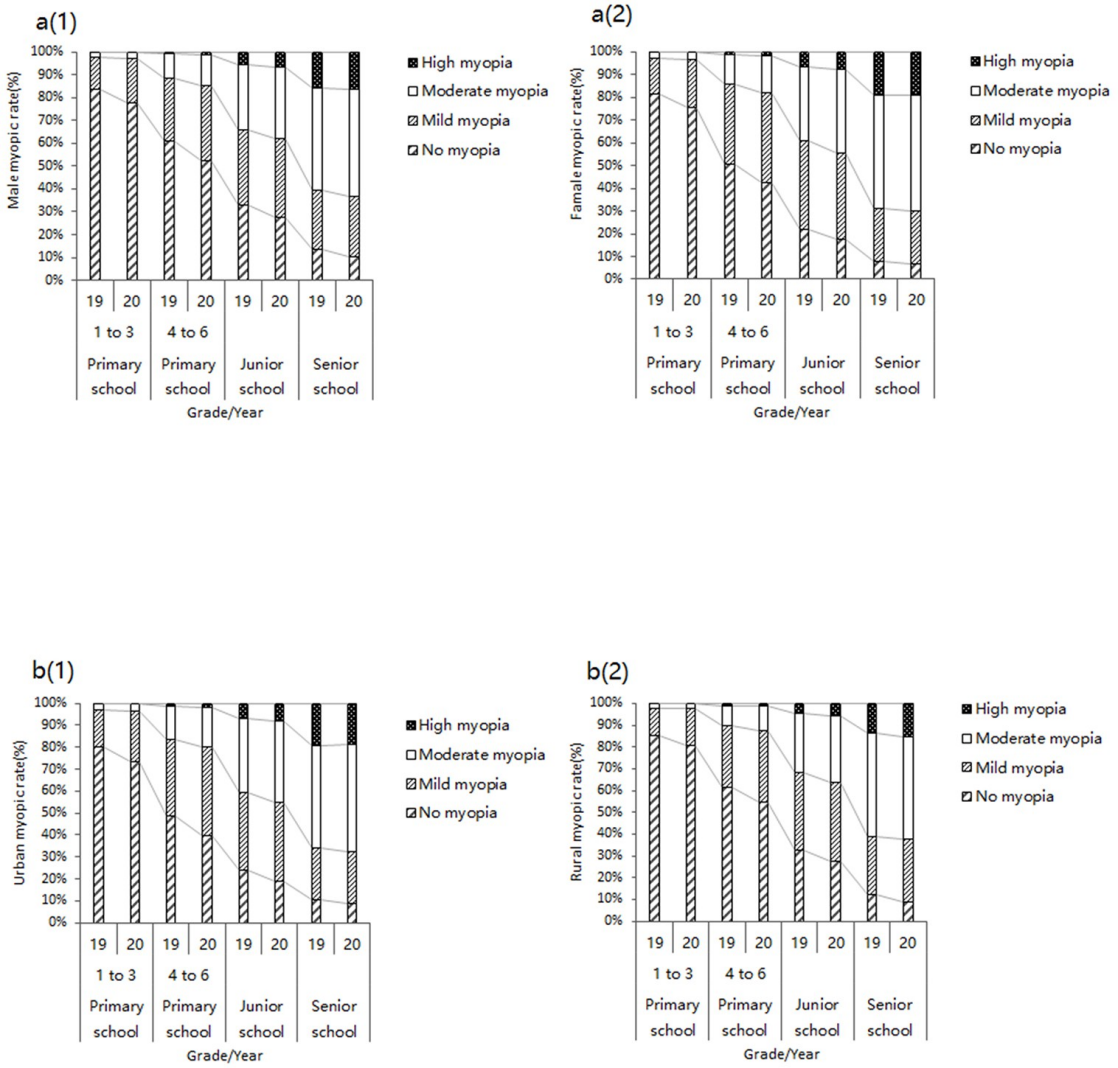
The distribution of different category of myopic prevalence in gender and region.

The different categories of myopic prevalence by sex and region are shown in Fig 1. The increasing trend of different degrees of myopia was clearly visible in 2020 compared to 2019, while the prevalence of myopia was increased in each grade in various genders and regions. The mild myopia had a wider distribution among primary and junior school students, while the moderate myopia showed higher prevalence among senior school students. In addition, there was a relatively lower prevalence of high myopia in rural areas.

### Refractive error

The mean SER of the right eye is reported in Table 3. The total SER in 2020 (-1.34±2.03 D) was less than that in 2019 (-1.16±1.92 D, *P*<0.05). Meanwhile, we found that the different demographic SERs in 2020 were less than those in 2019 (*P*<0.05), except for students aged 6, 16 to 18. In 2019 and 2020, the SER decreased with increasing age (*r*_2019_ = -0.51, *r*_2020_ = -0.51, *P*<0.01). The SER in girls (-1.25±1.97 D in 2019, -1.45±2.10 D in 2020) was less than that in boys (-1.08±1.87 D in 2019, -1.26±1.96 D in 2020, *P*<0.05), and was lower in urban students (-1.43±2.06 D, -1.59±2.13 D in 2020) than in rural students (-0.90±1.75 D, -1.09±1.88 D in 2020, *P*<0.05). The SER presented myopic status at the age of 9 (-0.55±1.26 D in 2019, -0.71±1.42D in 2020), and attained moderate myopia at the age of 15 (-3.06±2.41 D in 2019, -3.22±2.40 D in 2020).

**Table 3:** Demographic factors associated with SER of right eye in all participants.

	SER(D)		*t*	*P* ^e^
	2019	2020		
**All**	-1.16±1.92	-1.34±2.03	23.09	<0.01
**Age**				
**6**	-0.12±0.84	-0.10±1.54	-1.43	0.15
**7**	-0.16±0.85	-0.22±0.91	5.44	<0.01
**8**	-0.31±1.08	-0.42±1.14	7.58	<0.01
**9**	-0.55±1.26	-0.71±1.42	10.28	<0.01
**10**	-0.80±1.63	-1.05±1.61	13.24	<0.01
**11**	-1.18±1.76	-1.43±1.77	11.39	<0.01
**12**	-1.67±1.95	-1.83±2.02	6.26	<0.01
**13**	-2.12±2.05	-2.35±2.10	7.99	<0.01
**14**	-2.59±2.27	-2.77±2.29	5.28	<0.01
**15**	-3.06±2.41	-3.22±2.40	3.05	<0.01
**16**	-3.48±2.37	-3.59±2.38	1.82	0.07
**17**	-3.67±2.37	-3.73±2.38	0.91	0.37
**18**	-3.72±2.40	-3.78±2.67	0.45	0.66
***R***	-0.51	-0.51		
***P*** ^d^	<0.01	<0.01		
**Gender**				
**Male**	-1.08±1.87	-1.26±1.96	16.40	0.00
**Female**	-1.25±1.97	-1.45±2.10	16.17	0.00
***t***	15.48	16.70		
***P*** ^e^	<0.01	<0.01		
**Region**				
**Urban**	-1.43±2.06	-1.59±2.13	13.28	<0.01
**Rural**	-0.90±1.75	-1.09±1.88	17.40	<0.01
***t***	-46.90	-43.15		
***P*** ^e^	<0.01	<0.01		

Values are presented as mean ± SD.

^d^ Comparison underwent Pearson’s correlation analysis.

^e^ Comparison underwent Two-proportions z test.

## Discussion

This was a relatively comprehensive study of the prevalence of myopia in children and adolescents before and after the COVID-19 outbreak. As a cross-sectional study, there was a higher prevalence of myopia showing an increased trend with age and appearing in girls and urban students, which drew some similar conclusions as the previous study [21–23]. Meanwhile, mild myopia accounted for the largest proportion in primary school students. Moreover, it demonstrated that the prevalence of overall myopia in children and adolescents in 2020 increased by 5.8 percentage points compared to that in 2019. There was another lower result showing that the prevalence of myopia in Chinese students only increased by 2.5 percentage points during the COVID-19 pandemic [24]. Our study also showed the SER was more negative in 2020 than in 2019. Overall, we finally found myopic prevalence increased by year, which was in accordance with previous surveys [25–27]. It was speculated that competitive educational pressure accompanying rapid socioeconomic development in China may be one effective factor.

There have been several studies regarding the prevalence of myopia after the COVID-19 pandemic. A cross-sectional study about school-age students in Shandong, China [28], showed that comparing with 4 years before, the prevalence of myopia increased by 1.2–3 times after the COVID-19 pandemic, and it increased more among 6 to 8 year-olds. In a study conducted in Hong Kong, the annual incidence of myopia was 29.68% in the COVID-19 cohort compared with 11.63% in the pre-COVID-19 cohort [29]. A study in Chongqing, China [19], revealed that the prevalence of myopia increased from 45.3% in 2019 to 55.4% in 2020, and there was a greater increase among primary school students, compared with middle school students, which was in accordance with ours. Others had also mentioned that the age of 6–11 was the stage of rapid progression of myopia [30, 31]. There were some differences between other researches and ours. On the one hand, it may result from different regions and timing of home quarantines. On the other hand, the screening methods were also varied, such as the difference of refractive equipment. Indeed, there were studies reporting the different equipment could lead to varied results under noncycloplegic refraction [32, 33]. We did not investigate the living conditions of students during the pandemic. However, according to a Chinese survey on the living environment of students during home quarantine [34], students spent less time on outdoor activities and more time in a darker environment [4]. Due to the reduction of sunlight exposure would affect the secretion of dopamine in the eye, which could lead to the development of myopia [35]. The students in Suqian spent overall 2 months learning online, and it has been speculated to be a potential impact on the development of myopia.

We also found that myopia occurred in younger students, the SER presented negative at the age of 6, and appeared myopic status at the age of 9. Previous researchers also revealed this phenomenon. A similar survey in Taiwan demonstrated that myopia occurred at the age of 9 and moderate myopia occurred at the age of 18 in 1995 [36], moreover, they found the mean refractive index reached myopic status at the age of 8 in 2000 [37]. Shandong demonstrated myopia appeared at the age of 9 in 2015, and the diopter presented negative at the age of 6 in 2020, while the change in SER during the COVID-19 pandemic was more than that in the past 4 years [28]. A study of Turkey revealed that the decrease of SER in 2020 compared to the 2019 and 2018 years was significantly [38]. The younger trend of myopia onset also occurred in other Asian countries, such as Japan [39] and India [40], but it was not as obvious in Australia [41, 42] and Norway [43]. This phenomenon indicated that the prevalence of myopia was related to the different education and living conditions of various countries. Education pressure in Asian countries has made myopic status more severe [8, 44].

Although this study had a large sample size and high coverage, there were some potential limitations that should be mentioned. First, we only acquired the two years of data about the prevalence of myopia, so that we wouldn’t compare that with the previous conditions. Second, we did not investigate the living habits of students during the pandemic. Third, noncycloplegic refraction might lead to some errors, such as overestimation of myopia. In addition, a small number of students’ SER might be overestimated because of conducting refraction without taking off contact lenses, but the potential effect on this large sample size study was extremely low. In the future, we will design prospective studies to provide more reliable conclusions. We need to be alert about the occurrence and development of high myopia, because early myopia onset increases the risk of developing high myopia [45, 46].

## Conclusion

Our findings indicated that the myopic prevalence increased within various demographic characteristics after the COVID-19 pandemic, and the myopic shift in children was more than that in adolescents. It was possible that the primary school students were more sensitive to the changes of lifestyle than the middle school students. Additionally, myopia has occurred in younger students, and COVID-19 may have accelerated this phenomenon. Some strategies are necessary to adjust the living habits of lower grade students to delay the progression of myopia.

## Supporting information

S1 Data (XLSX)
